# Characterization of Anesthesia in Rats from EEG in Terms of Long-Range Correlations

**DOI:** 10.3390/diagnostics13030426

**Published:** 2023-01-24

**Authors:** Inna A. Blokhina, Alexander A. Koronovskii, Alexander V. Dmitrenko, Inna V. Elizarova, Tatyana V. Moiseikina, Matvey A. Tuzhilkin, Oxana V. Semyachkina-Glushkovskaya, Alexey N. Pavlov

**Affiliations:** 1 Department of Human and Animal Physiology, Saratov State University, Astrakhanskaya Str. 83, Saratov 410012, Russia; 2 Physics of Open Systems Department, Saratov State University, Astrakhanskaya Str. 83, Saratov 410012, Russia; 3 Regional Scientific and Educational Mathematical Center “Mathematics of Future Technologies”, Saratov 410012, Russia

**Keywords:** long-range correlations, scaling exponent, fluctuation analysis, electroencephalogram, anesthesia, diagnostics

## Abstract

Long-range correlations are often used as diagnostic markers in physiological research. Due to the limitations of conventional techniques, their characterizations are typically carried out with alternative approaches, such as the detrended fluctuation analysis (DFA). In our previous works, we found EEG-related markers of the blood–brain barrier (BBB), which limits the penetration of major drugs into the brain. However, anesthetics can penetrate the BBB, affecting its function in a dose-related manner. Here, we study two types of anesthesia widely used in experiments on animals, including zoletil/xylazine and isoflurane in optimal doses not associated with changes in the BBB. Based on DFA, we reveal informative characteristics of the electrical activity of the brain during such doses that are important for controlling the depth of anesthesia in long-term experiments using magnetic resonance imaging, multiphoton microscopy, etc., which are crucial for the interpretation of experimental results. These findings provide an important informative platform for the enhancement and refinement of surgery, since the EEG-based DFA analysis of BBB can easily be used during surgery as a tool for characterizing normal BBB functions under anesthesia.

## 1. Introduction

Natural systems often exhibit complex dynamics with long-range power–law correlations [1] that can be quantified by a slowly decaying correlation or covariation function. Such quantification is highly important for a deeper understanding of system behavior and the effects of long memory. In particular, it clearly differs from the exponentially decaying correlation function observed in the dynamics of deterministic models producing chaotic oscillation. The presence of power–law statistics is associated with the absence of a specific time scale and with the existence of the self-similarity property (a scale-invariant structure), when a wide range of scales takes part in the global description of the observed dynamics. Physiology in general shows many examples of systems, where power–law statistics (1/*f* activity) are combined with various rhythmic contributions, and numerous studies have been performed to explain the origin of the 1/*f* spectrum slope in recorded signals [2,3,4,5,6]. The classical correlation function has at least two restrictions in studying the effects of long memory, such as the difficulty in revealing its law of decay as values tend to zero, and its inapplicability to systems with time-varying parameters producing nonstationary behavior. In this regard, alternative signal processing tools for long-range correlation analyses have been developed, which include R/S analysis [7], different versions of fluctuation analysis [8,9], with the detrended fluctuation analysis (DFA) [10,11] as the most widely used technique, wavelet-based multifractal formalism [12,13], etc. Currently, DFA is a popular approach that has some limitations [14,15,16], but it is a fairly simple tool (compared, e.g., with the multifractal analysis [13]), which can better resolve the range of long-range correlations than the correlation function.

Scale-invariant structures of physiological processes often have distinctions between healthy and pathological states [17,18,19], as well as between various pathological states [20]. Due to this circumstance, a quantitative assessment of long-range correlations provides informative diagnostic measures. This quantification can be provided by the scaling exponent of DFA [10] that has a relation to the exponents describing the decay of the correlation function or the slope of the spectral power. However, in many cases, it is not enough to apply a single (global) quantity to describe correlations over the entire range of available scales. Thus, the paper [11] discusses the different scaling behavior for long-range and short-range correlations in heart rate dynamics. The latter means that the local scaling exponents can outperform the average measure in characterizing signal features. An alternative way is to consider the multifractal concept and quantify the temporal variations of the scale-invariant structure of physiological data sets in terms of a singularity spectrum, which can be estimated using the wavelet-transform modulus maxima method [12] or multifractal DFA [21,22]. Despite these tools providing informative and detailed characterizations of complex scaling in inhomogeneous physiological time series, even the simplest approach dealing with conventional DFA with the introduction of local slopes of fluctuations in the signal profile from a local trend depending on the scale parameter is a useful way for diagnostic-related studies.

Recent studies [23,24,25,26,27,28,29] showed that sleep and BBB openings are two conditions associated with similar activation of the brain’s drainage system. They proposed EEG markers for these states based on several signal processing techniques, including DFA and machine learning tools. In this work, we perform a DFA analysis of anesthesia because the BBB prevents the efficient delivery of major drugs into the central nervous system. However, anesthetics can penetrate the BBB to induce clinical anesthesia. If the optimal dose of anesthesia is applied, the permeability of the BBB is restored immediately after anesthesia is turned off, which is of high clinical relevance. For example, Spieth et al. [30] demonstrated that isoflurane causes a temporary opening of the BBB, and BBB functions were recovered directly after the termination of anesthesia, providing a certain window for drug delivery. The effects of anesthesia on BBB functions are highly dependent on the dose of anesthesia. For example, 1% isoflurane does not affect the BBB, while 2% and 3% induce an increase in the BBB permeability to high and small molecular weight substances [30,31,32,33]. Here, we study EEG characteristics in rats under two types of anesthesia at optimal doses widely used in animal experiments, including zoletil/xylazine (100 mg/kg/10 mg/kg, respectively) and 1% isoflurane, which are not associated with changes in the BBB [30,31]. We aim to develop a new method for EEG control of BBB permeability during surgical procedures and long-term experiments requiring the use of such types of anesthesia (magnetic resonance imaging, multiphoton microscopy, etc.), which is important for interpreting the experimental results. This study is also important for the enhancement and refinement of surgery because EEG-based DFA analysis of BBB can easily be used during surgery as a tool for characterizing normal BBB functions under anesthesia. In general, the effects of anesthesia on the body are widely known. It causes a loss of muscle tone and a related reduction in resting lung volume [34]. It affects the cardiovascular system and can produce a variety of cardiac depression and hemodynamic instability [35]. Anesthesia prevents communication between neurons in distinct areas of the brain [36], and the induced state of unconsciousness by destroyed coordinated neuronal processes in the central nervous system is different from sleep [37].

Here, we discuss how different types of anesthesia are reflected in power–law correlations in the EEG characterized by DFA local scaling exponents. The paper is organized as follows. Section 2 gives a brief description of the method for studying the correlation features of EEG signals and experiments in rats. Section 3 describes the main findings of the effects of anesthesia on the electrical activity of the brain and their discussion for both types of artificial sleep. Section 4 provides a summary of concluding remarks.

## 2. Methods and Experiments

### 2.1. Subjects

Experiments were carried out on male Wistar rats (2 months old) in accordance with the Guide for the Care and Use of Laboratory Animals published by the US National Institutes of Health (NIH Publication no. 85-23, revised 1996) and protocols approved by the Institutional Review Board of Saratov State University (Protocol 9, 26 June 2022). Animals were kept at a temperature of 25 ± 2 °C, humidity at 55%, and a light–dark cycle of 12:12 h. Food and water were given ad libitum. Rats were taken from the vivarium in Pushchino (Russia) one week before the start of the experiments to ensure acclimatization to the housing room. Experiments were performed on two groups of animals: (1) injection anesthesia with zoletil/xylazine (100 mg/kg/10 mg/kg, Virbac Sante Animale, France/NITA-FARM, Russia, respectively), and (2) inhalation anesthesia with 1% isoflurane at 1 L/min N${}_{2}$O/O${}_{2}$—70:30, (Dexa Medica, USA); *n* = 7 in each group. The design of experiments included EEG recording for 30–35 min without anesthesia; then the EEG was recorded for the next 30–40 min in the same animals that received anesthesia at the dose recommended for the surgery.

### 2.2. EEG Recording

Two-channel cortical EEGs [38,39] were recorded (Pinnacle Technology, Taiwan). Two silver electrodes (tip diameter 2–3 μm) were implanted into the frontal cortex to a depth of 150 μm in coordinates (L: 2.0 mm and P: 2 mm) from Bregma on either side of the midline under inhalation anesthesia with 1% isoflurane at a dose of 1 L/min N${}_{2}$O/O${}_{2}$—70:30. The head plate was mounted and small burr holes were drilled. Then, EEG wire leads were inserted into the burr holes on one side of the midline between the skull and underlying dura mater. The EEG leads were fixed with dental acrylic. Ibuprofen (15 mg/kg) to relive postoperative pain was given to them with the water supply two to three days before surgery and for three days after surgery. The rats were given 10 days to recover from the surgery before the start of the experiment.

### 2.3. DFA

A detrended fluctuation analysis was proposed [10,11] and widely used in various studies [40,41,42,43,44] as an alternative to the conventional correlation analysis of nonstationary processes and signals with a rapidly decreasing correlation function, which complicates the quantification of long-range correlations due to significant computational errors, especially for noisy data. This method represents a version of the root-mean-square analysis of random walks with a local detrending procedure. The DFA includes building a profile of a signal x(i),i=1,…,N as $y\left(k\right)=\sum_{i=1}^{k}x\left(i\right)$. This profile is separated into segments of length *n*. Depending on the amount of data, segments may or may not overlap. Profile fluctuations from the local trend $y_{n}\left(k\right)$ are computed and averaged over the entire data set y(k)

(1) $$F\left(n\right)=\sqrt{\frac{1}{N}\sum_{k=1}^{N}{\left[y\left(k\right)-y_{n}\left(k\right)\right]}^{2}.}$$

where $y_{n}\left(k\right)$ is fitted within each segment with the least-squares method using linear or nonlinear functions. The computations are carried out for a variable length of the segment *n* to analyze the power–law dependence

(2) $$F\left(n\right)∼n^{\alpha }.$$

In the presence of scale-invariant structures, the global quantity α can describe the features of power–law correlations in the signal x(i). In particular, it makes it possible to distinguish between anti-correlations (α<0.5), uncorrelated behavior (α=0.5), and positive correlations of various types (α>0.5). For complex signals produced by physiological systems, distinctions in the correlation features can depend on the scale and, therefore, a number of local scaling exponents can give a more complete description of the dynamics under study.

## 3. Results and Discussion

**Figure 1:**
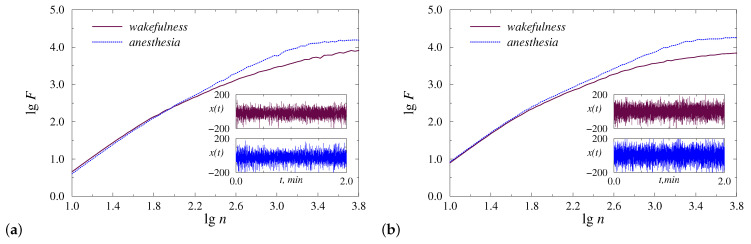
Examples of nonlinear behavior of F(n) on a log–log plot for injection (**a**) and inhalation (**b**) anesthesia. Insets show fragments of the original EEG signal from each condition.

The preliminary analysis of the recorded data sets to select an appropriate range of scales for computing the DFA scaling exponent shows nonlinear behavior of F(n) on a log-log plot (Figure 1). Although there are visual differences between the two states, namely wakefulness and anesthesia, they are not enough to use a single α-exponent for each state, and its local values become preferable. This circumstance is related to both types of anesthesia. The lgn regions associated with the strongest inter-state distinctions may differ depending on the animal and on the position of the electrode; therefore, it seems appropriate to establish the optimal values of the computational parameters for each record and then consider the statistics for the entire groups of animals. According to Figure 1, these optimal values belong to the region of relatively long-range correlations, and for lgn<1.8, the results are very close.

**Figure 2:**
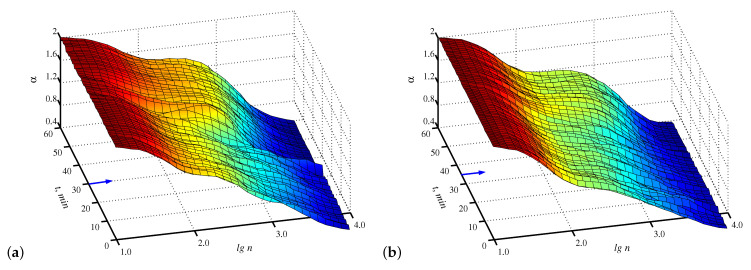
Examples of computations of local scaling exponents on a plane (time, range of scales) for injection (**a**) and inhalation (**b**) anesthesia. The arrows indicate when the anesthesia injection or inhalation started.

Figure 2 provides a fairly informative representation of computations on a plane (time, range of scales), that clearly shows the structural changes in signals caused by the transition to the anesthesia stage. Note that here we estimate scaling exponents locally, i.e., compute the local slopes of lgF versus lgn within a sliding window lgn of size 0.7. Figure 2 illustrates that despite the distinctions appearing after the implementation of anesthesia, the choice of an appropriate range of scales is of significant importance for diagnostic purposes. In particular, visually more essential differences take place near lgn=2.8 compared to other values of the lgn, and these distinctions are better identified in Figure 2a.

**Figure 3:**
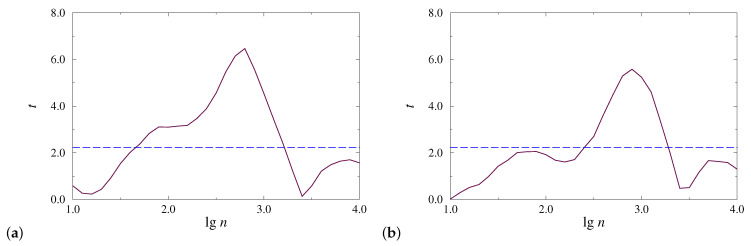
Estimates of *t*-values of the Student’s *t*-test for EEG signals for injection (**a**) and inhalation (**b**) anesthesia.

In addition to visual control of the results, they can be quantified on the basis of statistical tests. In order to compare diagnostic abilities, depending on the type of anesthesia, we estimated *t*-values of the Student’s *t*-test for EEG signals related to the two considered physiological conditions. For this purpose, we chose 10 EEG segments of 1 min each for wakefulness and 10 segments related to anesthesia. Figure 3 shows the performed estimates for the same records that were used for the computations given in Figure 1 and Figure 2. These estimates confirm the existence of an optimal lgn value that provides the strongest distinctions between physiological states, and give a range of scales providing reliable diagnostic results (*t*-values that go beyond the dashed line are associated with significant differences with p<0.05). In the considered examples, the optimal values of lgn are 2.8 (Figure 3a) and 2.9 (Figure 3b). The scale ranges that can be used to diagnose the effects of anesthesia are [1.7, 3.2] (Figure 3a) and [2.4, 3.2] (Figure 3b). Thus, in the examples considered, the first type of anesthesia caused stronger changes in EEG signals with larger *t*-values quantifying the differences between the states of wakefulness and anesthesia.

**Figure 4:**
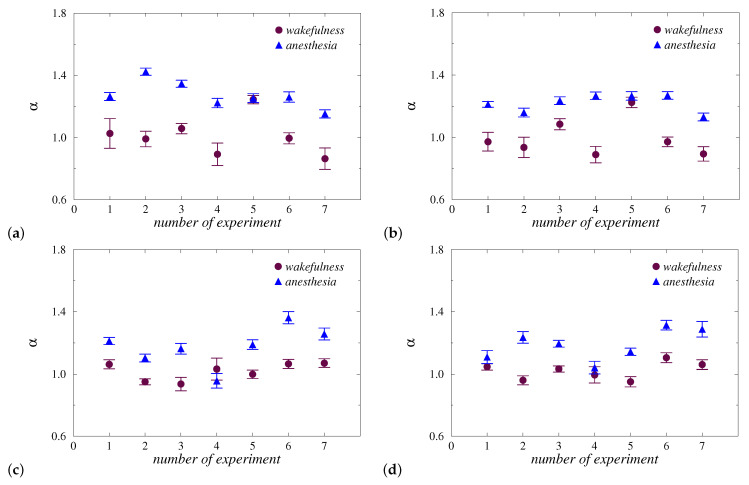
Local α-values for groups of animals for injection (**a**,**b**) and inhalation (**c**,**d**) anesthesia. Results are shown for the 1st (**a**,**c**) and 2nd (**b**,**d**) EEG channels.

The statistical analysis gives the optimal value of lgn equal to 2.6, as the average quantity for all groups of rats and all EEG-channels. Based on this assessment, local α-values related to the two states were compared for each type of anesthesia and EEG channel. The results shown in Figure 4 demonstrate similar distinctions. For both types of anesthesia, a reliable diagnosis of changes in long-range correlations was revealed. During injection anesthesia with zoletil/xylazine, 6 out of 7 animals showed an increase in the local scaling exponent, and the results for both channels were quite similar (Figure 4a,b). In particular, only for the 5th rat, the approach used did not reveal significant inter-state distinctions. For other animals, the changes in α-values significantly exceed the standard deviations of the estimated quantities. During inhalation anesthesia with isoflurane, the results are analogous (Figure 4c,d), namely, in 6 out of 7 rats, strong changes in α are observed when examining the 1st EEG channel (the absence of clear changes takes place only for the 4th rat). In the case of the 2nd EEG channel, a less clear reaction was also observed in the 1st rat.

Note that the diagnostic results given in Figure 4 were obtained by choosing lgn=2.6 as the optimal (on average) value of the scale parameter. If dealing with individually selected scales associated with the maximum *t*-value of the Student’s *t*-test, the separation of states can be improved. Table 1 contains the given estimates including the maximum *t*-value and the related scale range. For 27 out of 28 EEG signals, it becomes possible to identify changes in correlation features during the transition from wakefulness to anesthesia. Despite both types of anesthesia providing similar diagnostic results, they can nevertheless produce different structural changes in EEG signals. Thus, the first type of anesthesia often causes longer effects, and for the second type, such effects can be shorter.

**Figure 5:**
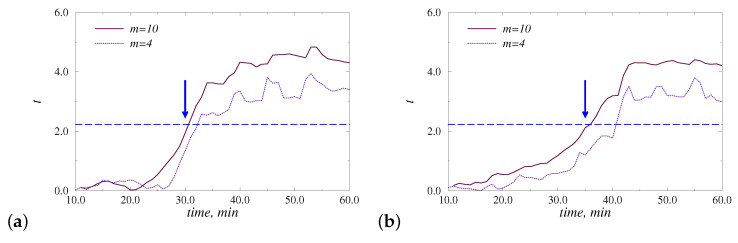
Examples of the *t*-values estimated by comparing *m* beginning 1-min EEG-segments with subsequent segments in a sliding window approach. The time of administration of anesthesia is marked with an arrow. Results are shown for injection (**a**) and inhalation (**b**) anesthesia. The dashed line indicates the critical value $t_{c}$ = 2.228 associated with *p* < 0.05.

In addition to the quantitative assessment of differences in steady-state regimes, structural changes in EEG signals were analyzed from transient processes after the administration of anesthesia. Figure 5 illustrates the *t*-values computed by comparing *m* = 10 EEG-segments related to the beginning 10 min of the awake state with subsequent data parts in a sliding window approach. Due to the averaging effect, transitions between states in *t*-values are quite slow. However, the crossing of the dashed line, indicating the critical value $t_{c}$ = 2.228 related to p<0.05, occurs close to the time of administration of anesthesia (marked with an arrow). Consideration of a smaller number of EEG segments (e.g., m=4 in Figure 5) does not essentially improve the identification of the corresponding transition between states.

## 4. Conclusions

Characterization of long-range correlations is often carried out for physiological systems to provide markers of changes in scale-invariant structures caused by transitions between distinct physiological states. The limitations of the traditional correlation function are the reason for the increased attention to alternative approaches, such as fluctuation analysis and DFA, as special cases. This method also has disadvantages, e.g., associated with a reliable quantitative assessment of signal features in the presence of non-stationarity and a trend without their prior exclusion. The latter can lead to incorrect conclusions about the underlying dynamics. Nevertheless, DFA is a useful approach for many studies, assuming that thorough signal preprocessing is carried out. The complex structure of the experimental time series may require careful data analysis using local scaling exponents that describe signal features in distinct scale ranges. Such type of analysis is performed in the current study by quantifying distinctions in EEG signals during anesthesia depending on the scale parameter and testing for significant changes in local scaling exponents using Student’s *t*-test.

Our analysis revealed significant distinctions in EEG in the state of wakefulness and anesthesia in terms of correlations in the range of lgn approximately [2.0–3.0]. These distinctions can be detected by choosing fixed algorithmic parameters, but the individual selection of such parameters, taking into account the features of each record, makes it possible to improve the diagnostic capabilities of the method. Thus, the use of two features of the DFA method, namely, local scaling exponents and individual adjustment of algorithmic parameters that maximize Student’s t-values, provided a reliable identification of the effects of anesthesia in 27 out of 28 records. Such identification gives similar results for both types of anesthesia, namely injection and inhalation anesthesia, although the first type often causes prolonged effects in terms of long-range correlations. Consideration of transient processes allows one to correctly detect transitions between states, regardless of the type of anesthesia. These results provide an important informative platform for the enhancement and refinement of surgery since the EEG-based DFA analysis of BBB can easily be used during surgery as a tool for characterizing normal BBB functions under anesthesia.

## Figures and Tables

**Table 1:** The maximum *t*-value and the optimal scale range for individual records and both types of anesthesia.

Rat Number	EEG-Channel	Maximum *t*-Value	Optimal lgn
Injection anesthesia			
1	1	3.41	3.4
	2	3.85	2.5
2	1	7.14	2.6
	2	4.27	2.0
3	1	6.47	2.8
	2	3.61	2.0
4	1	4.76	2.7
	2	6.49	2.4
5	1	2.24	3.1
	2	2.31	3.1
6	1	4.67	2.7
	2	6.86	2.3
7	1	5.29	2.9
	2	4.72	2.9
Inhalation anesthesia			
1	1	4.16	2.1
	2	3.78	2.0
2	1	3.22	2.6
	2	5.56	2.5
3	1	3.52	2.8
	2	2.54	2.6
4	1	2.68	2.3
	2	1.46	2.9
5	1	6.94	3.0
	2	5.58	2.9
6	1	5.24	2.7
	2	4.04	2.7
7	1	5.40	2.8
	2	6.27	2.9

## Data Availability

The data that support the findings of this study are available from the corresponding author upon reasonable request.

## Abbreviations

The following abbreviations are used in this manuscript:

BBB	blood–brain barrier
DFA	detrended fluctuation analysis
EEG	electroencephalogram
